# Automatic detection of pleural line and lung sliding in lung ultrasonography using convolutional neural networks

**DOI:** 10.1016/j.heliyon.2024.e34700

**Published:** 2024-07-24

**Authors:** Takeyoshi Uchida, Yukimi Tanaka, Akihiro Suzuki

**Affiliations:** a Material Strength Standards Group, Research Institute for Engineering Measurement, National Metrology Institute of Japan, National Institute of Advanced Industrial Science and Technology, Central 3, 1-1-1 Umezono, Tsukuba, 305-8563, Japan; b Department of Anesthesiology and Critical Care Medicine, Jichi Medical University, Yakushiji, Shimotsuke-shi, Tochigi, 329-0498, Japan

**Keywords:** Ultrasound diagnosis, Lung ultrasonography, Pleural line, Lung sliding, Pneumothorax, Convolutional neural network, Lung lesions

## Abstract

**Background:**

Lung ultrasonography (LUS) is a valuable diagnostic tool, but there is a shortage of LUS experts with extensive knowledge and significant experience in the field. Convolutional neural networks (CNNs) have the potential to mitigate this issue by facilitating computer-aided diagnosis.

**Methods:**

We propose computer-aided system by a CNN-based method for LUS diagnosis. As the first consideration, we investigated pleural line and lung sliding. The pleural line indicates the position of pleura in an ultrasound image, and LUS is performed after first confirming the position of pleural line. Lung sliding defined as the movement of the pleural line, and the absence of this feature is associated with pneumothorax.

**Results:**

Our proposed method accurately detected pleural line and lung sliding, demonstrating its potential to provide valuable diagnostic information on lung lesions.

## Highlights

• A computer-aid diagnosis system based on image recognition using convolution neural networks
• Our computer-aid system was developed to address the shortage of medical experts in lung ultrasonography.
• The system can automatically detect the pleural line and lung sliding in lung ultrasonography images.
• The system has potential for utilization as a tool in clinical practice.

## 1 Introduction

Ultrasonography, employing ultrasonic diagnostic equipment in the medical field is a safe and convenient diagnostic tool that has recently expanded as point-of-care ultrasonography [1,2]. Advances in ultrasound technology, including device miniaturization, digital technology integration, and enhanced image quality, have significantly improved the ability to observe tissue structures in detail [3,4]. As a result, lung diagnostics via ultrasonography, known as lung ultrasonography (LUS), have become feasible [5,6].

LUS has emerged as a relatively new diagnostic modality, gaining widespread use over the past decade [7]. Ultrasound waves are reflected at the border between the air in the lung and the visceral pleura due to differences in their specific acoustic impedances [8]. Consequently, LUS usually uses movement of visceral pleura and other artifacts to diagnose lung lesions such as pneumothorax, pneumonia.

Traditionally, chest radiography is used to diagnose lung lesions [9]. For pneumothorax diagnosis, LUS has higher sensitivity than chest radiography [[10], [11], [12]]. Also, LUS is advantageous for routine application because it is safe, convenient, cost-effective, and does not require special installation. The COVID-19 pandemic has further highlight the benefits of LUS [13]. Nevertheless, LUS demands specialized ultrasound knowledge, specific diagnostic protocols, and extensive diagnostic experience. The training of LUS experts is time-and cost-intensive, and leading to a chronic shortage of skilled practitioners in clinical practice. This shortage has increased the workload per LUS expert and reduced diagnostic efficiency, particularly during the COVID-19 pandemic, the lack of LUS experts has resulted in reduced utilization of LUS [14].

To address these challenges, this study demonstrates the effectiveness of convolutional neural networks (CNNs) in enhancing LUS diagnostics. CNNs, deep learning method in artificial intelligence (AI), have been extensively used in many studies for medical diagnosis, condition analysis, and patient healthcare [[15], [16], [17], [18]].

Our objective was to leverage CNN-based analysis of ultrasound images about lung lesions to improve diagnostic efficiency and accuracy. For the first consideration, we focused on detecting pleural line and lung sliding, fundamental features in LUS. This led to the development of AI systems capable of identifying challenging diagnostic features. The pleural line is an important feature to be first identified in LUS. The pleural line typically consists of three structures: the visceral pleura, the parietal pleura, and the physiological pleural fluid that lies between them [5]. Distinguishing these structures can sometimes be challenging. In the context of LUS, this is observed at the depth of the posterior aspect of the ribs as part of the characteristic bat sign. Lung sliding represents the movement of pleural line with respiration. The normal lung shows lung sliding, a horizontal movement with respiration at the pleural line, indicating sliding of the visceral pleura against the parietal pleura. However, in cases of pneumothorax, only the parietal pleura is visualized, and lung sliding is absent.

## 2 Methods

**Fig. 1:**
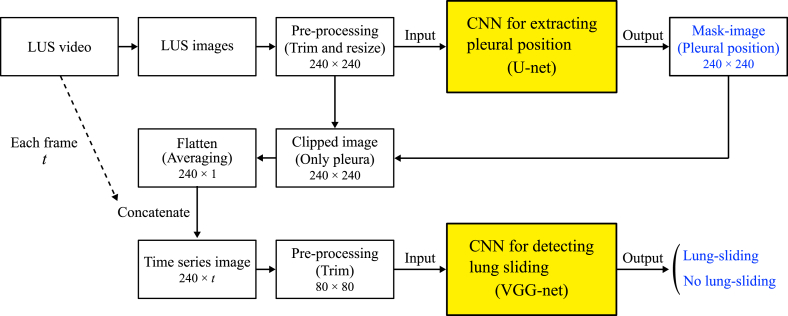
Block diagram of CNN system for pleural line and lung sliding.

**Fig. 2:**
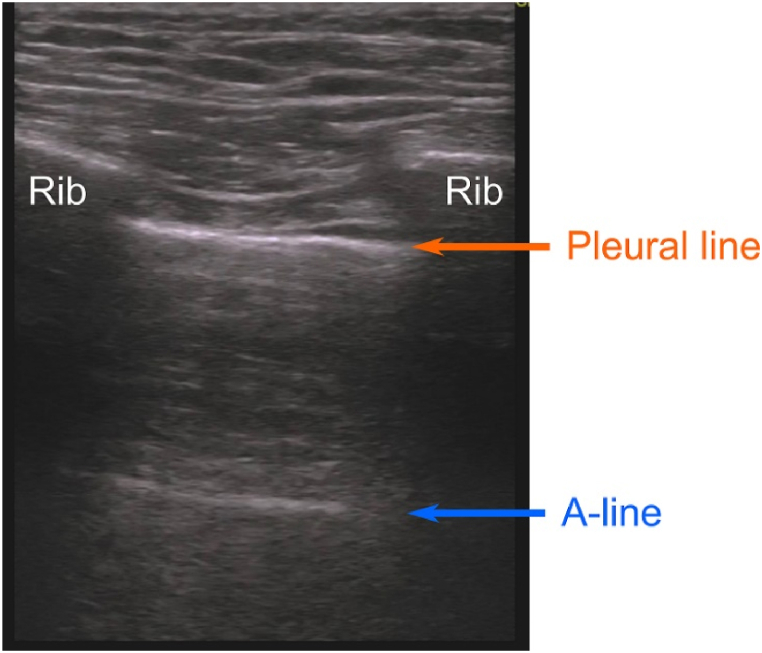
Pleural line in LUS.

Fig. 1 shows a block diagram of the automated detection system used to detect lung features from ultrasound images obtained using CNNs. This section describes the process of automatic detection of pleural line and lung sliding using CNNs, as shown in Fig. 2. The lung ultrasound images were trimmed and resized to a 240 × 240-pixel grayscale version of the original high-resolution image. We used two CNNs; the first CNN (U-net) extracted the pleural region from the LUS images, whereas the second CNN (VGGNet) detected lung sliding from time-series images generated according to the mask images of the pleural position from the U-net [19]. The following sections present details of the CNN structure.

### 2.1 Automatically detection pleural line using CNNs

#### 2.1.1 U-net for detecting pleural line

**Fig. 3:**
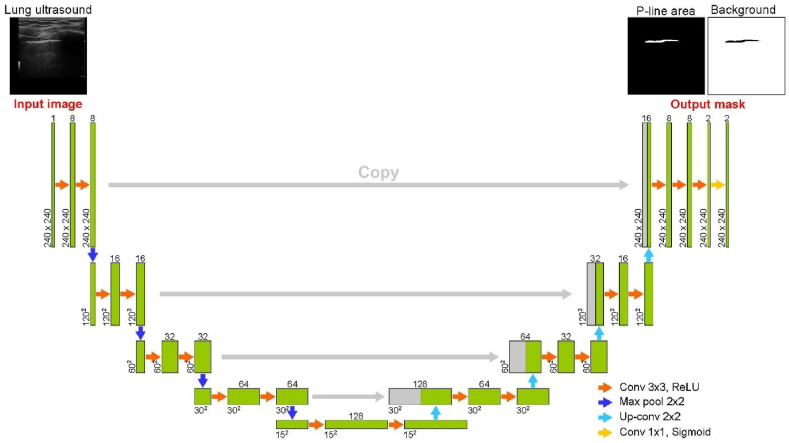
Structure of U-net.

In this experiment, we utilized U-net, a CNN model designed for semantic segmentation. U-net is adept at identifying features of lung lesions based on the pixels in medical images. We implemented U-net using Python and Keras with a TensorFlow backend to detect pleural line (Fig. 3). The input image to U-net model was a lung ultrasound image with a resolution of 240 × 240 pixels. The output images were two mask images, each with the same dimensions as the input image, representing the pleural line area and its background. U-net comprises two main components: a contracting path and an expansive path. The contracting path, which extracts essential features, follows the conventional U-net structure. It includes two 3 × 3 convolutional layers followed by 2 × 2 max pooling for downsampling. The expansive path consists of upsampling via a 2 × 2 up-convolution, which reduces the number of feature channels by half. This path also involves concatenation with corresponding cropped feature map from the contracting path and two 3 × 3 convolutional layers. Rectified linear units were employed as activation functions after each convolution in both the contracting and expansive paths. To ensure rapid training and to prevent overfitting, batch normalization was applied in each convolution [20].

#### 2.1.2 Dataset for U-net training

We prepared a dataset of human lung ultrasound images to train the U-net model, which we subsequently validated. This dataset comprised ultrasound images serving as input for the U-net, and corresponding mask images delineating the pleural line and background regions. The ultrasound images of human lungs were captured by ultrasound medical specialists at Jichi Medical University Hospital using GE, Venue50 portable ultrasound diagnostic equipment with linear and convex probes operating at 11 MHz. Subsequently, these lung ultrasound images were processed by trimming and resizing them to a 240 × 240-pixel grayscale format, retaining the essential details from the original high-resolution images. To create the mask images, which highlight the region recognized by ultrasound medical specialists, we manually delineated the pleural line using ImageJ software. The background of the mask images was generated by inverting the area outside the pleural line. In total, 10,000 ultrasound images were prepared, and mask images were created for the training dataset. To augment the training dataset, we employed image processing techniques to increase the number of images. This involved randomly changing the image position, angle, contrast, and adding noise to the original images. Additionally, we prepared a validation dataset comprising 140 ultrasound images, which remained unaltered to assess the CNNs performance.

All procedures were performed in accordance with the ethical standards of the Responsible Committee on Human Experimentation (institutional and national guidelines) and with the Helsinki Declaration of 1964 and later versions. The study was approved by the Institutional Review Board for Clinical Research, Tokai University (ID: 21R-048) on 11 June 2021. Written informed consent was obtained from all participants prior to their inclusion in the study.

#### 2.1.3 Training and evaluating U-net

To mitigate overfitting and underfitting, we assessed the training accuracy of the U-net model. The training of U-net indicated that the weight values were optimized to reduce the difference, called the loss function, between the output and reference positions of the pleural line. We employed the dice score coefficient to quantify the similarity between two images, thereby evaluating the segmentation performance. Notably, the U-net architecture utilized the dice loss, a function derived from the dice score coefficient, for semantic segmentation [21]. Mathematically, the dice loss, $L_{dice}$, can be expressed as

(1) $$L_{dice}\left(g,p\right)=1-\frac{2\sum_{i}^{N}p_{i}g_{i}}{\sum_{i}^{N}p_{i}+\sum_{i}^{N}g_{i}+1}\text{,}$$

where *p*_*i*_ presents the predicted binary segmentation volume, and *g*_*i*_ denotes the ground truth binary volume. The minimization of this loss function was achieved through a mini-batch gradient descent (with a mini-batch size of 32), and a learning rate set to 0.0001. Training proceeded until the loss function reached a sufficiently low and stable level, with U-net trained for 100 epochs.

The performance evaluation of U-net typically employed the *Dice coefficient* (*DC*) and *Jaccard similarity coefficient* (*JSC*) [22]. These metrics were utilized to assess the similarity between the pleural line determined by ultrasound medical specialists and the U-net outputs. The *DC* was computed as:

(2) $$DC=\frac{TP}{TP+\frac{1}{2}\left(FP+FN\right)}\text{,}$$

where *TP*, *FP*, and *FN* represent the number of pixels in each category as delineated in Table 1. Furthermore, the *DC* serves as the harmonic mean of recall and precision. A *DC* value of 1 signifies accurate prediction, with values approaching 1 reliable pleural line prediction.

**Table 1:** Definition of abbreviations.

Category	Actual pleural line	Actual no pleural line
Predicted pleural line	*True positive* (*TP*)	*False positive* (*FP*)
Predicted no pleural line	*False negative* (*FN*)	*True negative* (*TN*)

*JSC* was calculated as:

(3) $$JSC=\frac{TP}{TP+FP+FN}\text{.}$$

where *TP*, *FP*, and *FN* are as shown in Table 1.

A higher *JSC* indicates greater overlap between predicted and ground truth results, with a perfect prediction yielding a *JSC* of 1.

*DC* and *JSC* values were computed using both the training (utilized for U-net training) and validation datasets (not employed in U-net training), facilitating a comprehensive evaluation of U-net performance.

### 2.2 Experiment to automatically detect lung sliding using CNNs

#### 2.2.1 VGGNet for detecting lung sliding

**Fig. 4:**
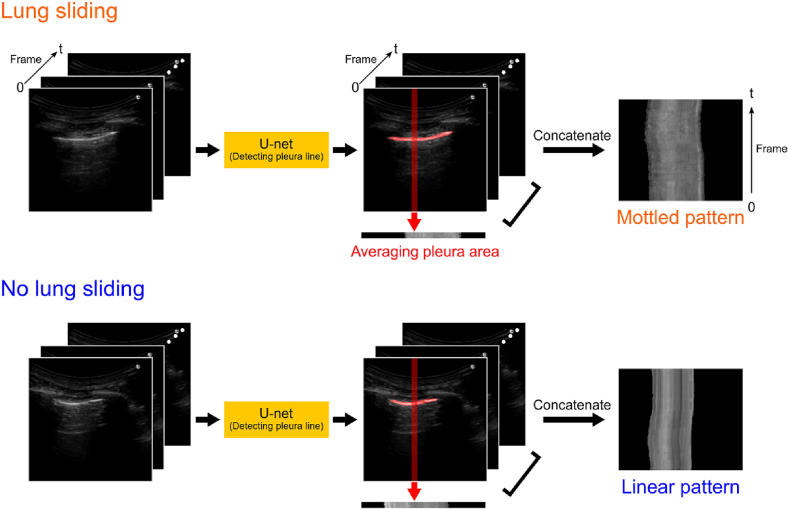
Time-series image of the pleural line.

To detect gradual changes in the pleural line, video data were essential. The 2D-CNN method was employed in this study to capture alterations in the pleural line over time and detect lung sliding (Fig. 4). During the thoracotomy procedure for lung surgery, a video clip was recorded and saved. This clip clearly shows the deflated lung on the surgical field monitor, allowing for the differentiation between pneumothorax state and normal states. Utilizing U-net, images of the pleural area were extracted from each frame of the video data, as shown in Fig. 1. These images were converted into one-dimensional images by averaging the pixel values along the depth direction. Subsequently, time-series images were obtained by arranging these one-dimensional images vertically according to their chronological order. The presence of lung sliding was indicated by a mottled pattern in the time-series image, whereas its absence resulted in, a linear pattern. These time-series images were served as inputs for the CNNs, and the output indicating the presence or absence of lung sliding.

**Fig. 5:**
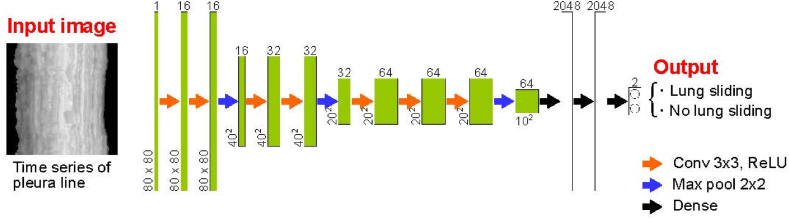
Structure of 2D-CNN.

Python and Keras with a TensorFlow backend were utilized to implement VGGNet for the detection of lung sliding. The CNN structure based on the VGGNet architecture [23] is illustrated in Fig. 5. Time-series images of dimensions 80 × 80 pixel served as input for the VGGNet with the output representing the probability of lung and non-lung sliding within the range of 0–1. The VGGNet architecture consists of seven convolutional layers, three maximum pooling layers, and two fully connected layers. The convolutional filters were sized at 3 × 3 with a stride of 1 and, 2 × 2 max pooling was performed with a stride of 1. Each fully connected layer comprised 2048 neurons. Both normalization was applied to each convolutional layer to facilitate rapid training without overfitting [20]. Additionally, to prevent overtraining, a dropout (with a drop rate of 0.5) was implemented between the fully connected layers.

#### 2.2.2 Dataset for VGGNet training

A dataset was compiled specifically for training the VGGNet on lung sliding images. This dataset consisted of 5601 images (time-series images, as shown in Fig. 5), each paired with corresponding labels indicating either “lung sliding” or “no lung sliding". To augment the training data for VGGNet, the image count was increased by introducing random alterations to the image position, angle, contrast, and noise in comparison to the original images. Additionally, 2000 time-series images were prepared to assess the performance of VGGNet.

#### 2.2.3 Training and evaluating VGGNet

Binary cross-entropy loss, a widely used loss function for binary classification [22] was employed to determine the area under the curve (AUC), serving as the evaluation metric for VGGNet. The following equation represents the loss function suitable for the lung-sliding experiment, denoted as *L*_*BCE*_:

(4) $$L_{BCE}\left(y,\hat{y}\right)=-\left(y\;\log\hat{y}+\left(1-y\right)\log\left(1-\hat{y}\right)\right)\text{,}$$

where $\hat{y}$ is the predicted value, and y is the true value. The loss function was optimized using mini-batch gradient descent with a mini-batch size of 32, and fixed learning rate of 0.0001. Training proceeded until the loss function reached a sufficiently low and stable state, spanning 100 epochs.

The AUC can be used to evaluate the classification performance of machine learning models [23,24]. A value closer to 1, signifies the superior performance of the CNN. To obtain the AUC, a receiver operating characteristic (ROC) curve was generated, which illustrates the relationship between the true positive rate (TPR) and the false positive rate (FPR). TPR denotes the percentage of correctly predicted instances, while FPR represents the percentage of incorrectly predicted instances. ROC curve and its corresponding AUC were computed using the validation dataset.

## 3 Results and discussion

### 3.1 Pleural line

**Fig. 6:**
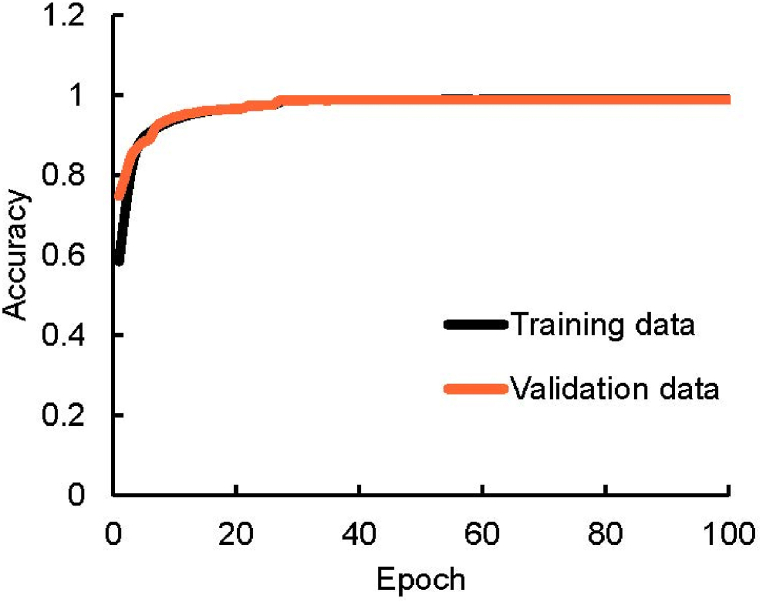
Relationship between epoch value and accuracy of CNN based on ultrasound diagnosis images of human lungs.

**Fig. 7:**
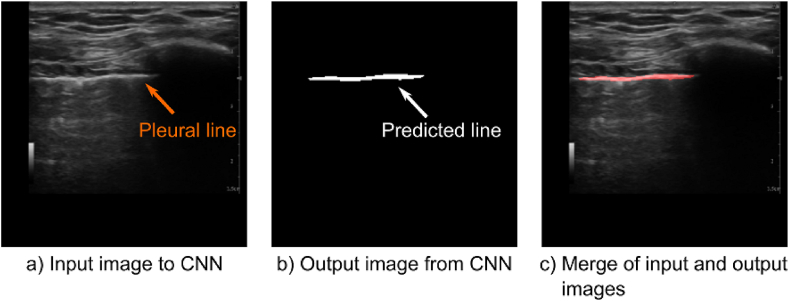
Example of reference and predicted line images of pleural line. a): input image to CNN; b): output image from CNN; c): merge of input and output images.

To ensure the performance of the U-net mitigate overfitting or underfitting, training accuracy was assessed, as shown in Fig. 6. The results from both training and validation datasets approached 1 at 100 epochs, indicative of minimal overfitting or underfitting. Post-training, the performance of the U-net in pleural line, extraction was evaluated. Fig. 7 showcases samples of reference and predicted pleural line images. The alignment of pleural line in Fig. 7(a) and (b), as shown in Fig. 7(c), underscores the precision of the U-net in predicting the pleural line.

Additionally, the performance of the U-net was quantified using *DC* and *JSC* yielding values of 0.9878 and 0.9762, respectively. Given their proximity to 1, it can be inferred that the pleural line was accurately detected within the dataset comprising ultrasonic diagnostic lung images, underscoring the potential of the U-net for automated detection.

**Fig. 8:**
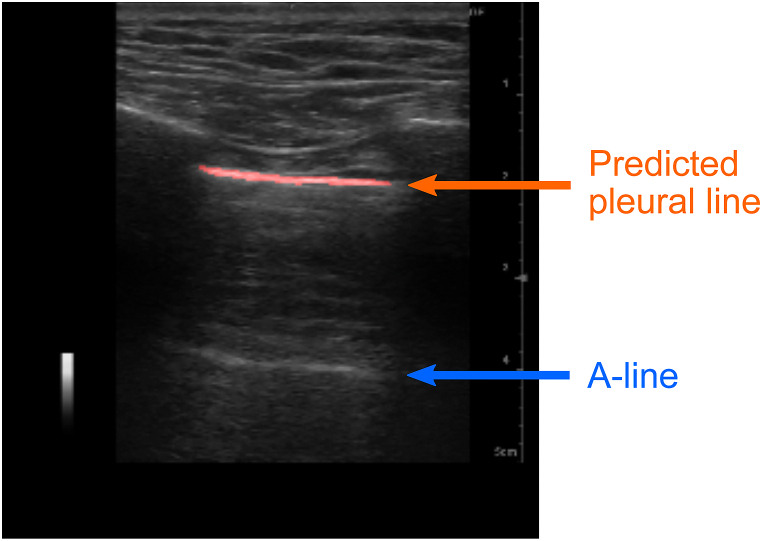
Example of distinguishing between pleural line and A-line.

The U-net has the potential that pleural line and A-line can be automatically distinguished as shown in Fig. 8. A-line manifests as a horizontal artifact denoting reverberation, arising from ultrasound wave reflection between ultrasound probe surface and air in lung. This phenomenon occurs in both healthy, air-filled lung and pneumothorax cases, where air prompts strong ultrasound beam reflection to the probe. General image processing struggles to differentiate between the pleural line and A-line due to their akin luminance values, shapes, and sizes, leading to diagnostic confusion by providing AI information. Our U-net demonstrated potential in distinguishing between these lines, paving the way for future investigations into A-line evaluation.

**Fig. 9:**
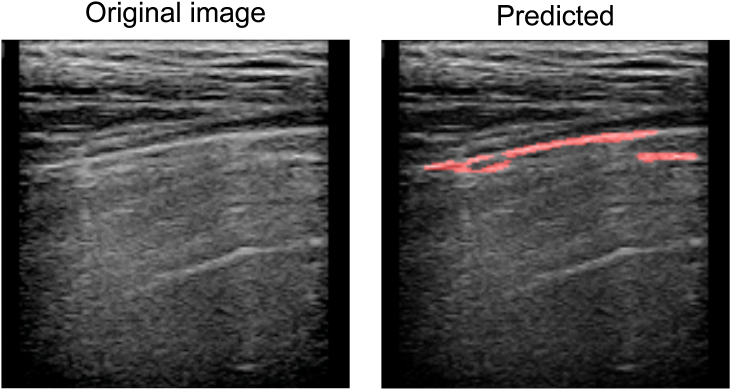
Example of false detection of pleural line.

In contrast, while U-net demonstrated commendable performance in terms of *DC* and *JSC*, instances of false detections were sporadically noted, as shown in Fig. 9. Specifically, lines comprised of multiple echoes originating from the fascia were erroneously identified. The misidentified lines exhibited similar positional and brightness characteristics to those of the pleural line, posing a challenge for the U-net in distinguishing between them. It is conceivable that these artifacts were erroneously classified as pleural line. Subsequent investigations will endeavor to enhance the accuracy of pleural line detection by augmenting the volume of training data utilized.

### 3.2 Lung sliding and lung point

**Fig. 10:**
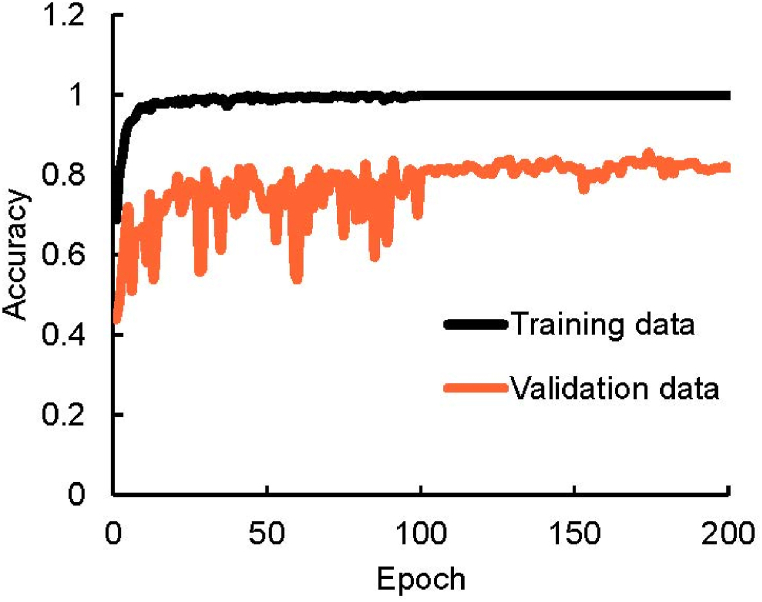
Relationship between epoch value and accuracy of CNN for lung sliding.

**Fig. 11:**
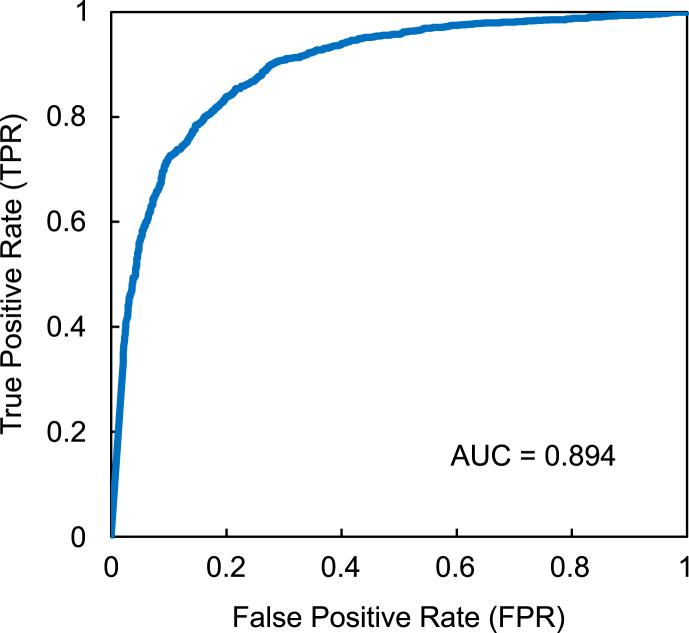
ROC curve of CNN for lung sliding.

In the lung sliding experiment, we conducted a comparative analysis of the training and validation data results to mitigate issues related to overfitting and underfitting during the VGGnet training process. A key metric for assessing this was the proximity of these results to a value of 1, indicating the optimal balance for efficient training without overfitting or underfitting (Fig. 10). The training data result closely approached 1, while the validation data result was approximately 0.8 after 200 epochs. This indicates that neither overfitting nor underfitting occurred, confirming the efficacy of our training approach. The ROC curves are illustrated in Fig. 11 with an AUC value of 0.894. This value demonstrates that the CNNs exhibited a high level of accuracy in detecting the presence or absence of lung sliding.

**Fig. 12:**
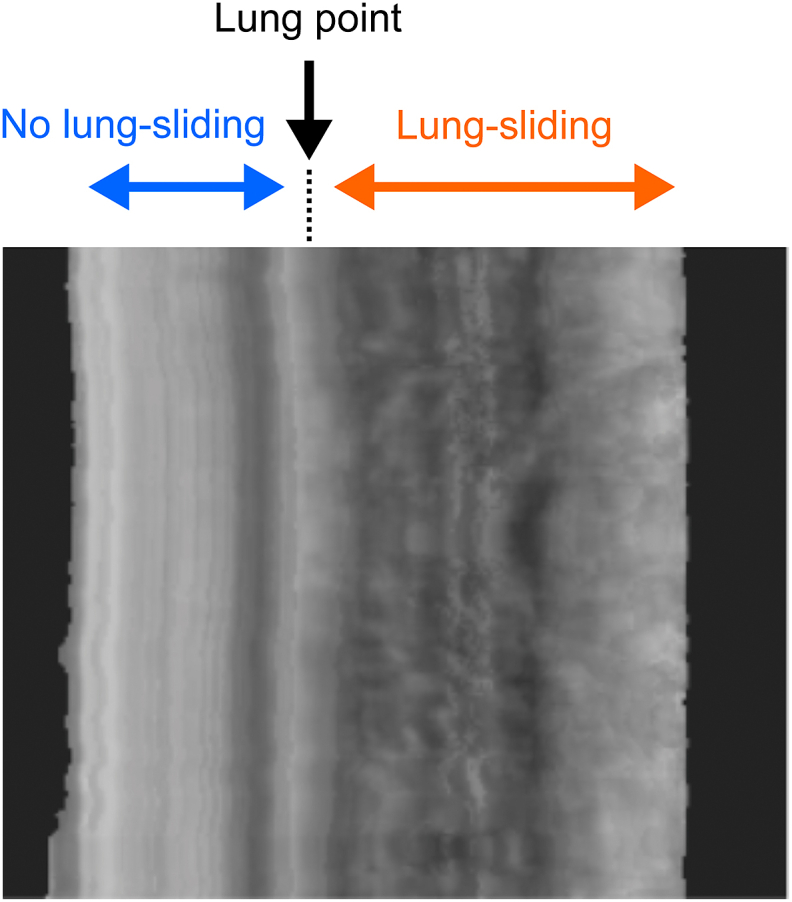
Example of prediction on lung point by CNNs.

The detection of lung sliding is generally performed using B-mode ultrasound. Recently, it has been advocated to employ both B-mode and M-mode in tandem, as this combined approach enhances diagnostic precision [25,26]. The proposed VGGnet method has the potential to detect the lung sliding in a single measurement with high accuracy because it is possible to automatically use time variation image like M-mode image in addition to B-mode image. As lung point which is a pathognomonic sign of the presence of a pneumothorax, increase of diagnosis accuracy can be expected because our VGGnet method use the time variation of entire pleural region which is easy to judge lung point as shown in Fig. 12. The lung point serves as a crucial reference for delineating the vertical demarcation between a mottled pattern, indicative of lung sliding, and a linear pattern, denoting its absence.

### 3.3 Perspective of CNN method

Our method demonstrated good performance in automatically detecting pleural line and lung sliding. There are already a few reports on diagnostic methods for LUS, but there are few on the reports on automatic detection by CNNs. Our results have proved the effectiveness of image recognition in LUS using a CNN-based segmentation method. This observation can be attributed to the relatively advanced diagnostic protocol of LUS compared to other organ-specific diagnostic modalities.

Currently, there is no consensus on the integration of CNNs into daily clinical practice. CNNs predictions cannot supplant the expertise of medical specialists unless achieving 100 % reliability. The system evaluated in this study demonstrates the potential utility of CNNs in capturing fundamental LUS features. However, its clinical applicability remains limited at present. Nevertheless, as the system keeps on evolving, it holds promise for furnishing diagnostic insight in clinical settings. Furthermore, it can serve educational purposes, mitigating the shortage of ultrasound medical specialists by facilitating comprehension of complex lung lesion-related information by non-LUS specialists.

As with the majority of studies, the design of our investigation is subject to certain limitations. Primarily, the accuracy of our CNN-based technique was contingent upon training data acquired under controlled conditions. Secondary, the technique was tailored to discern only rudimentary LUS features. Consequently, its performance might diminish in real-world clinical practice. Nonetheless, in terms of image analysis by CNNs, it is important to perform basic study on ultrasound images which are difficult to analyze images and leading to practical use in the future. We are currently investigating the performance of our CNNs under difficult conditions, for example, cases of the patient showing breathing difficult and pneumothorax under subcutaneous emphysema.

## 4 Conclusions

This study investigated the effectiveness of CNNs for LUS. We developed an automatic detection system for identifying pleural line and lung sliding in LUS utilizing a segmentation method. Our CNN-based system can automatically extract pleural line appropriately and detect lung sliding with high accuracy using extracted pleural regions. The proposed method will contribute to the provision of diagnostic information on lung lesions.

In future research, we aim to evaluate additional LUS features such as A-line and lung point, and explore the automatic detection of B-line [27], which are significant indicators for pneumonia, pulmonary edema, and lung contusion. B-lines manifest as vertical artifacts originating from the pleural line. Furthermore, we intend to assess to the impact of ultrasound exposure conditions such as operating frequency and probe form, on changes in AUC.

## Data availability statement

No data associated in this article has been deposited into a publicly available repository. Data will be made available on request.

## Funding

None.

## CRediT authorship contribution statement

**Takeyoshi Uchida:** Writing – review & editing, Writing – original draft, Visualization, Supervision, Project administration, Investigation, Formal analysis, Data curation, Conceptualization. **Yukimi Tanaka:** Writing – review & editing, Investigation, Formal analysis, Data curation, software. **Akihiro Suzuki:** Writing – review & editing, Resources.

## Declaration of competing interest

The authors declare that they have no known competing financial interests or personal relationships that could have appeared to influence the work reported in this paper.
